# Using GIS-based methods of multicriteria analysis to construct socio-economic deprivation indices

**DOI:** 10.1186/1476-072X-6-17

**Published:** 2007-05-14

**Authors:** Nathaniel Bell, Nadine Schuurman, Michael V Hayes

**Affiliations:** 1 Department of Geography, Simon Fraser University, 8888 University Drive, Burnaby, British Columbia, Canada; 2 Faculty of Health Sciences, Simon Fraser University, 8888 University Drive, Burnaby, British Columbia, Canada

## Abstract

**Background:**

Over the past several decades researchers have produced substantial evidence of a social gradient in a variety of health outcomes, rising from systematic differences in income, education, employment conditions, and family dynamics within the population. Social gradients in health are measured using deprivation indices, which are typically constructed from aggregated socio-economic data taken from the national census – a technique which dates back at least until the early 1970's. The primary method of index construction over the last decade has been a Principal Component Analysis. Seldom are the indices constructed from survey-based data sources due to the inherent difficulty in validating the subjectivity of the response scores. We argue that this very subjectivity can uncover spatial distributions of local health outcomes. Moreover, indication of neighbourhood socio-economic status may go underrepresented when weighted without expert opinion. In this paper we propose the use of geographic information science (GIS) for constructing the index. We employ a GIS-based Order Weighted Average (OWA) Multicriteria Analysis (MCA) as a technique to validate deprivation indices that are constructed using more qualitative data sources. Both OWA and traditional MCA are well known and used methodologies in spatial analysis but have had little application in social epidemiology.

**Results:**

A survey of British Columbia's Medical Health Officers (MHOs) was used to populate the MCA-based index. Seven variables were selected and weighted based on the survey results. OWA variable weights assign both local and global weights to the index variables using a sliding scale, producing a range of variable scenarios. The local weights also provide leverage for controlling the level of uncertainty in the MHO response scores. This is distinct from traditional deprivation indices in that the weighting is simultaneously dictated by the original respondent scores and the value of the variables in the dataset.

**Conclusion:**

OWA-based MCA is a sensitive instrument that permits incorporation of expert opinion in quantifying socio-economic gradients in health status. OWA applies both subjective and objective weights to the index variables, thus providing a more rational means of incorporating survey results into spatial analysis.

## Background

Research on health and place has produced substantial evidence that living in places with higher relative measures of socio-economic deprivation has a negative influence on a variety of health outcomes [1]. A key observation in population health research is that inequalities in health are linked to systematic differences in social class/socio-economic position [2,3]. Gradients in health are routinely associated with the cumulative effect of employment circumstances and working conditions, poverty, educational attainment, early development and several other social factors. [3-8]. The logic of using deprivation indices to estimate relative health status is based on this regularly occurring pattern of population health outcomes [9,10].

Rarely, however, are social gradients in health quantified using survey-based data sources. Deprivation indices are most frequently constructed using variations of either Principal Component or Factor Analysis – both of which are highly computational structural detection and data reduction strategies designed to reduce the number of variables needed to measure socio-economic status (SES). However, these strategies minimize the opportunity to incorporate the knowledge of local health practitioners and of the day-to-day conditions that impact local health outcomes. This is problematic as the underlying conditions that influence neighbourhood variations in SES may go underrepresented when assessed without using their local knowledge. Previously, the author's had constructed a survey-based deprivation index for use in British Columbia using feedback from provincial Medical Health Officers (MHOs) [11]. However, many questions remain as to the most appropriate way in which to measure stakeholder continuity. This paper demonstrates that GIS-based multicriteria analysis (MCA) techniques can be used to strengthen the value of deprivation indices constructed from qualitative data sources. Our analysis uses an Order Weighted Average (OWA) weighting algorithm, which was selected based on its ability to represent both the original and a data-driven ranking of the variables selected by the MHOs.

### Deprivation indices: a concise review

The most relied on source for areal estimation of population SES is the national census. Researchers often rely on the national census for socio-economic information about the population because these datasets are freely available (or available at a low cost), broadly representative of all political jurisdictions, and contain a number of variables reflective of an individual's or area's socio-economic position relative to the surrounding population. They also have pragmatic value as aggregate data is used in absence of individual data to protect anonymity. Census-based deprivation indices date back to the early 1970's in the UK and have since been developed in Canada, the US, New Zealand, and elsewhere [12-16].

There are three primary methods to quantify the effect of living under adverse socio-economic conditions. All three strategies are presented alongside their corresponding variable and scale components in table 1. The first is to create standardized percentages of the census indicators through numerator and denominator rates. Mapping raw rates, either through the use of standardized z-scores or log transformations, was the principal method of index construction in the UK up until the late 1980's [7,17]. The benefit of this approach is the ability to combine disparate indicator categories (i.e. cost with percentage) and the reduction of skewed data distributions. The standardized variables can then carry proportional weights relative to their importance in determining SES.

**Table 1:** Structure of six deprivation indices (^†^UK based; * Canadian based).

**Measure**	**Jarman**^†^	**Carstairs**^†^	**Townsend**^†^	SEFI*	DIHWPQ*	GDI*
**Type of Index**						
Material Deprivation	***X***	***X***	***X***	***X***	***X***	***X***
Social Deprivation	***X***	***X***		***X***	***X***	***X***

**Categories of Variables Used**						
Income variables		***X***		***X***	***X***	***X***
Housing variables	***X***	***X***	***X***			***X***
Demographic variables	***X***			***X***	***X***	***X***
Mobility variables	***X***	***X***	***X***			
Education variables				***X***	***X***	***X***
Employment variables	***X***	***X***	***X***	***X***	***X***	***X***

**Variable Weighting Method**						
Principal Component Analysis				***X***	***X***	***X***
Log transformations			***X***			
Expert Weighting	***X***					

**Geographic Unit of Analysis**						
Wards	***X***	***X***	***X***			
Enumeration/Dissemination Areas					***X***	
Census Tracts						***X***
Municipal boundaries				***X***		

The second technique for index construction is the use of either Principal Component or Factor Analysis – both of which have been the principal method of index construction over the past two decades [18-20]. PCA/FA are designed to reduce the complexity of large datasets by identifying the principal SES indicators that explain the underlying correlation within the entire dataset, ultimately replacing the original variables with a smaller number of components which then are used to measure SES. This strategy not only eliminates the need to assign *apriori *weights to the index variables, but also provides a medium to compress individual socio-economic variables into their underlying social (i.e. family dynamics) or material (i.e. educational attainment) constructs. However, in compressing a set of *n *variables into a smaller number of components the resulting index explains only a partial amount of the variance exhibited in the entire dataset and so some information regarding the original correlation between the variables is removed.

Although less popular than the preceding strategies, a third approach is to construct the index using feedback from health experts. The most widely used deprivation index built on survey-based data was the Jarman UPA8 index [21,22]. The UPA8 was first constructed as a workload assessment, and later as a payment formula, for British General Practitioners to help overcome the challenges embedded in a homogeneous capitation allowance. The score was constructed using a 10% sampling frame of British GP's, who were asked to comment on the factors that increased their daily stress and workload [23]. The score was based on eight of the most popular variables selected by the experts. All variables were obtained from the UK Census and weights were assigned to the index variables based on the frequency of the responses.

Ensuing critique over the UPA8 reliance on the census; its geographic preference for London over the Northern Regions; and its weighting of the survey scores have since curtailed widespread popularity of survey-based deprivation indices [24-26]. As a result, modern deprivation indices favour an approach based on Principal Component Analysis, due largely in part to the elimination of *apriori *weighting and the relative ease of constructing highly computational measures of deprivation using a wide number of statistical software packages (i.e. SPSS^©^, SAS^©^). In other research areas, however, integrating the day-to-day knowledge of local health experts using survey-based approach is seen as a viable means for uncovering local SES conditions [27]. In this paper we introduce MCA as an alternative to traditional deprivation index construction that utilizes a survey-based format. The addition of the MCA model is seen here as a means to strengthen analyses that are constructed from the point of view of multiple stakeholders.

### Multicriteria analysis

GIS-based MCA is one of the most common functions of geographical analysis but has not yet been thoroughly tested in social epidemiology – despite being introduced by Schuurman [28]. In geographical analysis MCA is typically used for the resolution of site suitability conflicts [29-31] and balancing the tradeoffs and risks associated with various expert opinions engaged in public policy implementation [32-34]. In many instances, these are overlapping and even conflicting environments [35,36].

The purpose MCA is to condense complex problems involving multiple criteria (e.g. variables) into an optimal ranking of the best variable scenarios from which an alternative is chosen [30,37-40]. In a GIS, this might involve a set of geographically defined criteria, such as soil grade, commercial or residential zoning, or hypsography and deriving alternatives for land use development based on the spatial arrangement of areas that meet the minimum or maximum of each of the evaluation criteria. Weights can be assigned to the criteria according to the importance of each variable in deriving the alternative and each combination of the variables and their weights may have a more or less favourable influence on the final decision than another.

The OWA approach is among many possible techniques for ranking multiple criteria, which range from simple scalar methods to complex models based on fuzzy logic [37,40-42]. OWA-based MCA developed out of the need to address uncertainty when modeling the interaction between multiple criteria. At the time of its inclusion in GIS, the two most popular functions of MCA were Boolean overlay and Weighted Linear Combination techniques. However, each technique was fundamentally flawed as they were poorly suited for the critical examination of underlying variable relationships. The OWA model is a more robust extension of these older MCA modeling approaches. Its principal advantage over the former techniques was the addition of a continuous scaling component set against the Boolean *union *(risk seeking) and *intersection *(risk adverse) operators. The scaling operator makes use of a pair of weights, one local, and one global. The local weights are assigned before hand and can be based on value judgments, proportional ranking, ratios, etc. The global weights are incrementally added and removed from one or all of the variables until reaching a full trade-off of equal weights on to the *n *variables. The order weights assigned between the full *union *and *trade-off *model favour a more risk-seeking modeling scenario, assigning larger weights to the highest ranking variables and lower weights to the smallest variables. The order weights assigned between the full *intersection *and *trade-off *model favour a more risk-adverse modeling scenario, where weights are maximized onto the variables with the smallest values and minimized on variables with higher initial values. The order weights are also assigned on a case-by-case basis. This allows the data to weight themselves according to their value relative to the other variables. The weighting flexibility of the OWA modeling environment is a robust tool for assessing how certain variables may compensate for other variables as weights are maximized, minimized, or allowed to trade-off equally. From a population health standpoint, this type of modeling framework may provide researchers a new vantage point from which to assess the SES indicator relationships (i.e. education and home ownership; lone parent and income).

## Methods

In this paper, we introduce an MCA model of socio-economic deprivation derived from a previous survey of British Columbia's MHOs. We employ 2001 Canadian Census data for the population of the Vancouver Census Metropolitan Area (CMA) to estimate SES. The utility of MCA is in synthesizing the variables and the weights assigned by the MHOs into a single, quantifiable model reflective of the socio-economic variables chosen by the experts, their weight, and the rank importance of their value existent in the dataset.

### Study site: the Vancouver Census Metropolitan Area

In 2001, the Vancouver CMA contained 21 municipalities and just under 2 million people, which is over half of the total population of the province. The municipalities in the Vancouver CMA encompass some of the most and least privileged neighbourhoods in all of Canada. The West Point Grey, Shaughnessy, and Kitsilano neighbourhoods, which are some of the wealthiest neighbourhoods in the country, closely border the Downtown Eastside, Oppenheimer, and Chinatown neighbourhoods, widely considered amongst the poorest. Researchers have long investigated the impact of social gradients in health within a number of municipalities in the urban core and the outlying suburban regions of the Fraser Valley [2,43-45]. However, a comprehensive index based on data from the census to measure intra-urban variations in socio-economic inequality throughout metropolitan Vancouver or other urban areas throughout British Columbia has yet to be developed. This research draws on the expert knowledge of the provincial MHOs to help populate a deprivation index for all of the municipalities within the Vancouver CMA.

### Data sources for self-rated health and socio-economic statistics

At the time of this analysis the province was still without a census-based construct specifically designed to measure health and socio-economic deprivation of British Columbian's. Rather than construct an index based on combining numerous census indicators into a principal component analysis we chose to survey provincial Medical Health Officers. We selected the MHOs because they are trained physicians with an interest in public health and well being. Part of the job requirement of provincial MHO's is also to trace disease/outbreaks and they have the responsibility, in terms of their own agency, to provide direction or oversight in assisting those in the community.

A complete description of the web survey and the particular variables selected for the analysis can be found elsewhere [11]. Briefly, the web-survey was originally distributed to the provincial MHOs between the months of June and August, 2005. All18 of British Columbia's MHOs were invited to complete the survey. Respondents were contacted via e-mail and their e-mail addresses were obtained from the chief Medical Health Officer. Each MHO was asked to rank a total of 21 census variables that they most strongly felt influenced socio-economic deprivation and relative health outcomes within urban areas in British Columbia. Each indicator had been previously employed in deprivation studies in Canada and abroad. Our goal was to include variables that we felt pertinent to residents in British Columbia as well as indicators from in previous studies and let the MHOs decide which ones were most relevant. The questions were close ended and responses were presented using a Likert scale (strongly agree – strongly disagree). The survey was organized into seven constructs which can be defined as broadly representing the conditions that tend to reflect *material wealth, housing tenure, family demographics, mobility, educational attainment, employment*, or *cultural identity*.

The material wealth component was constructed from two census variables, including average income and average dwelling value. These variables represent a direct and indirect measure of material standing. Average income is perhaps the most robust factor representative of material deprivation and is widely used as an indicator of socio-economic position. Similarly, dwelling value is a multidimensional indicator of purchasing power and has previously been found to positively co-vary with health outcomes in British Columbia [2]. *Housing tenure *included four indirect measures socio-economic position, including the percentage of single-detached housing units, the proportion of renters and owners, and those residing in an apartment. These variables broadly measure similar socio-economic conditions that were previously found to be associated with health outcomes in Vancouver [2]. Each of the six variables assigned to the family demographics construct has previously been linked to long-term health outcomes [46,47] and included elderly 65 and over and living alone, living alone, single-parent family, being single, divorced, or widowed, the number of persons under the age of five, and household overcrowding (family sizes greater than 5 persons). Both five year and 1 year movers from previous residence were included in the mobility construct. Similar variables have previously been used to measure social cohesion and neighbourhood stability [48,49]. The census variables in education construct broadly reflect both social and material deprivation as an individual can have a low income but still be regarded in higher esteem given their level of education. Both high school and university educational attainment rates were included in the education component. The employment construct contained three widely used indicators of socio-economic position both in Canada and abroad, including; the employment ratio, the unemployment rate, and the proportion of females in the labour force (as a measure of social exclusion). The cultural identify construct represented conditions that may act as barriers to obtaining goods and services or seen as a measure of social exclusion and included; non-Canadian citizens and the percentage of the population whose first spoken language was neither English nor French.

MHO responses to each of the 21 indicators were assigned a score between 1 and 5 (Strongly Agree = 5 to Strongly Disagree = 1). From the original survey, seven indicators were selected for the final index based on the proportion of the MHOs agree and strongly agree responses, including; average income, home ownership, lone parent family, having secondary and post secondary education, the employment ratio and unemployment rate. This subset was selected by administering a cut-off score to only include variables that had summation values greater than 'neutral' response plus 1, which symbolized all respondents choosing a non-neutral positive response for that particular variable. The original and final indicators and their proportional weights are listed in table 2.

**Table 2:** The global weights were constructed from the original MHO responses from the web-survey (SA = strongly agree; A = agree; N = neither agree/disagree; D = disagree; SD = strongly disagree).

Indicator Variables	BC Medical Health Officer Responses										Selected	Weight
	**I**	**II**	**III**	**IV**	**V**	**VI**	**VII**	**VIII**	**IX**	**X**		

***Material Wealth***												
*Average Income*	*A*	*A*	*A*	*SA*	*SA*	*A*	*A*	*A*	*N*	*SD*	*Yes*	0.089
*Average Dwelling Value*	*A*	*A*	*D*	*A*	*D*	*D*	*D*	*N*	*N*	*D*	*No*	
***Housing***												
*Single-detached Housing*	*N*	*A*	*N*	*N*	*D*	*N*	*D*	*N*	*N*	*SA*	*No*	
*Home Ownership*	*A*	*A*	*A*	*A*	*A*	*A*	*N*	*N*	*SA*	*SA*	*Yes*	0.089
*Proportion of Renters*	*A*	*A*	*A*	*A*	*A*	*N*	*A*	*N*	*SA*	*SA*	*No*	
*Reside in an Apartment*	*N*	*N*	*D*	*N*	*D*	*N*	*D*	*N*	*N*	*SA*	*No*	
***Demographics***												
*Elderly 65+ Living Alone*	*A*	*D*	*N*	*A*	*D*	*SA*	*N*	*SA*	*SA*	*SA*	*No*	
*Living Alone*	*N*	*D*	*A*	*A*	*N*	*A*	*A*	*A*	*A*	*SA*	*No*	
*Single Parent Family*	*A*	*SA*	*A*	*SA*	*A*	*SA*	*SA*	*N*	*A*	*N*	*Yes*	0.143
*Separated/Divorced/Widowed*	*A*	*N*	*N*	*A*	*D*	*A*	*A*	*N*	*N*	*N*	*No*	
*Children Under 5*	*D*	*N*	*N*	*N*	*D*	*A*	*A*	*N*	*SA*	*SA*	*No*	
*Family Size + 5 Persons*	*D*	*N*	*N*	*A*	*N*	*N*	*N*	*N*	*SA*	*N*	*No*	
***Mobility***												
*Moved in the Last 5 Years*	*N*	*N*	*D*	*A*	*N*	*N*	*A*	*A*	*N*	*N*	*No*	
*Moved in the Last Year*	*N*	*N*	*N*	*A*	*N*	*A*	*A*	*A*	*A*	*N*	*No*	
***Education***												
*No High school Completion*	*A*	*SA*	*SA*	*SA*	*A*	*A*	*SA*	*A*	*SA*	*SA*	*Yes*	0.250
*with a University Degree*	*SA*	*SA*	*N*	*SA*	*A*	*N*	*SA*	*A*	*A*	*SA*	*Yes*	0.179
***Employment***												
*Employment Ratio*	*SA*	*SA*	*D*	*SA*	*N*	*N*	*A*	*A*	*A*	*SA*	*Yes*	0.036
*Unemployment Rate*	*SA*	*SA*	*A*	*SA*	*N*	*SA*	*SA*	*A*	*A*	*SA*	*Yes*	0.214
*Females in Labour Force*	*N*	*A*	*N*	*A*	*N*	*N*	*A*	*N*	*A*	*SA*	*No*	
***Other***												
*Non-Canadian Citizen*	*D*	*N*	*N*	*N*	*D*	*A*	*A*	*N*	*A*	*SA*	*No*	
*First Language non Official*	*D*	*N*	*N*	*N*	*D*	*A*	*A*	*N*	*N*	*A*	*No*	

Health data were obtained from the Canadian Community Health Survey 2.1 (CCHS), a nation wide cross-sectional health survey of the population designed to allow comparison of health outcomes at a sub-provincial level across Canada. Data for the Cycle 2.1 database were collected between January and November of 2003. The target population of the CCHS is Canadians over 12 years of age who live in private dwellings. Individuals living on Indian Reserves, Crown Lands, institutional residents and full-time members of the armed forces are excluded. Data were collected primarily by telephone using three sampling frames, 48% from an area frame, 50% from a list frame of telephone numbers and 2% from random digit dialling. In our study, we use a sub-set of the CCHS response scores of respondents in the Vancouver CMA between the ages of 18 and 74 (n = 6,157).

Self-rated health data was assessed from the CCHS question "In general, would you say your health is: Excellent, Very Good, Good, Fair, Poor." Proxy measures of health status, such as self-rated health, are widely used as surrogate measures of population health and have shown a significant relationship with levels of mortality, morbidity and health care utilization [50,51]. For our analysis, we dichotomized the self-rated health into a *good health *component comprising the 'Excellent, Very Good or Good' responses and a *poor health *component comprising responses of fair or poor.

Confidence intervals for the prevalence estimates of the population reporting fair or poor self-rated health by neighbourhood SES quintile were obtained using 500 bootstrap weights provided by Statistics Canada using SAS software. The bootstrap weights are used to account for the complex design of the CCHS sampling frames. Sample weights were assigned to the self-rated health responses so that results were representative of the population living within the Vancouver CMA. The coefficients of variation (CV) produced using the bootstrapping weights were used to gauge the quality of estimates between the self-rated health responses and SES quintiles. The extent of the sampling error from the CCHS survey questionnaire is measured using the CV. The CV tables are prepared by Statistics Canada. Estimates less than 16.5% are deemed acceptable, estimates between 16.6% and 33.3% are flagged as marginal and estimates greater than 33.3% are flagged, but not released.

### Using OWA to build a deprivation index

Similar to traditional MCA spatial analysis, the OWA index was built from a series of overlay operations. Areal data obtained from the Canadian Census is geographically bound to set administration zones, which at the local level can range in size at the local level from Dissemination Area (DA), which are roughly the size of neighbouring neighbourhood blocks, to the municipal subdivision (CSD). Census DA boundaries were chosen for this analysis as they provide the finest lens to examine neighbourhood homogeneity. In the 2001 Canadian Census, the DA boundaries replaced the Enumeration Area (EA) as the basic unit of geographic dissemination. Each DA contains a target population ranging from 400 to 700 persons.

The OWA weighting logic is employed here to assess the degree to which the original weights assigned by the MHOs provides a more robust indication of neighbourhood SES than when local, data-driven weights are assigned to the indicator variables. The first set of weights assigned to the indicator variables are the global weights. These are universal weights (e.g. income is assigned the same weight throughout the entire study area) and represent the original ranked importance of the indicator variables assigned by the MHOs. The local weights are assigned on a case-by-case basis, where each case is represented by a unit of the census geography. The local, or *order weights*, are not assigned to the index variables according to the MHO ranking, but rather according to each indicator's position relative to the other variables in the dataset. Table 3 provides an example. In scenario 1, income is the variable exerting the least amount of influence on area SES in DAUID59150001 relative to education and housing and in a full Boolean *intersection *(⋂) model would carry 100% of the local weights. In DAUID59150002, however, income exerts the most influence on area SES. In this instance the variable representing education would receive 100% of the local weights. A number of weighting scenarios can be constructed using the OWA model, ranging from the classical risk-adverse Boolean *intersection *model to the risk-seeking Boolean *union *(⋂) model, with the classical weighted linear combination found in between. The benefit of this strategy is that the indicators most reflective of each area's SES position relative to the surrounding area are allowed to influence its rank. This enables the model to exhibit less influence from the MHO global weights, but also provides researchers with a theoretical base to assess the rank importance of the indicator variables selected by the MHOs.

**Table 3:** The local weights are assigned on a case-by-case basis relative to the indicator's value in the dataset. Table adapted from Malczewski [39]

**DAUID 59150001**				**DAUID 59150002**			
	Scenario 1 w^a ^= [1,0,0]				Scenario 2 w^a ^= [1,0,0]		
			
**Census Indicator**	**x**_i_	**w**^a^	**Value**	**Census Indicator**	**x**_i_	**w**^a^	**Value**
	
Income	0.33	1	0.33	Income	0.65	0	0
Education	0.42	0	0	Education	0.41	1	0.41
Housing	0.51	0	0	Housing	0.55	0	0
	Scenario 1 w^b ^= 0.5,0.3,0.2				Scenario 2 w^b ^= 0.5,0.3,0.2		
			
**Census Indicator**	**x**_i_	**w**^b^	**Value**	**Census Indicator**	**x**_i_	**w**^b^	**Value**
	
Income	0.33	0.5	0.17	Income	0.65	0.2	0.13
Education	0.42	0.3	0.13	Education	0.41	0.5	0.21
Housing	0.51	0.2	0.10	Housing	0.55	0.3	0.17

	Scenario 1 w^c ^= [0,0,1]				Scenario 2 w^c ^= [0,0,1]		
			
**Census Indicator**	**x**_i_	**w**^c^	**Value**	**Census Indicator**	**x**_i_	**w**^c^	**Value**
	
Income	0.33	0	0	Income	0.65	1	0.65
Education	0.42	0	0	Education	0.41	0	0
Housing	0.51	1	0.51	Housing	0.55	0	0

Table 4 lists the local, or order weights assigned to the seven indicator variables. The full *intersection*, or AND (⋂) OWA model assigns an order weight of 1 to the index variable within each Census unit that produces the lowest area score of the seven factors and a 0 to the remaining variables. In the full *intersection *model each Census unit is uniquely evaluated according to the least deprived of the seven factors within its own geography. The full *intersection *model may highlight severely deprived neighbourhoods much the same as a traditional deprivation index (e.g. areas experiencing multiple SES deprivation), but is designed to assess the strength of the SES variable relationships with a greater degree of control of the global weights assigned by the MHOs [37,39]. The antipode of the full *intersection *model is the Boolean *union*. The full *union*, or OR (⋃) OWA model assigns an order weight of 1 to the index variable that produces the highest area score of the seven factors and a 0 to all subsequent variables. This is similar in scope to the UK indices. Here, the deprivation score is assigned to census areas to maximize the level of deprivation within each area, although with some alteration as the full OR (⋃) model is represented by a single socio-economic variable, which implies *single *versus *multiple *deprivations. The remaining order weights are scaled across all SES variables between the crisp Boolean ANDing and ORing operators [30]. A full trade-off (averaging) of the order and local weights is obtained when all seven indicators are assigned order weights of equal value, which produces SES deprivation scores nearly synonymous to the original variable ranking assigned by the MHOs. It remains to be tested if a type of functionality that does not maximize the level of associated risk between socio-economic variables is the most beneficial in population health studies.

**Table 4:** Outline of the local (order) weights assigned to index variables.

**Operator**	**Order Weights**	***ANDness***	***Orness***	***Trade-Off***
*Full *Intersection	1, 0, 0, 0, 0, 0, 0	*1*	*0*	*0*
*AND *(b)	0.7, 0.15, 0.1, 0.05, 0, 0, 0	*0.92*	*0.08*	*0.35*
*AND *(c)	0.4, 0.25, 0.15, 0.1, 0.05, 0.025, 0.025	*0.78*	*0.22*	*0.63*
Trade Off (Ave)	0.142, 0.142, 0.142, 0.142, 0.142, 0.142, 0.142	*0.5*	*0.5*	*0.95*
*OR *(c)	0.025, 0.025, 0.05, 0.1, 0.15, 0.25, 0.4	*0.22*	*0.78*	*0.63*
*OR *(b)	0, 0, 0, 0.05, 0.1, 0.15, 0.7	*0.08*	*0.92*	*0.35*
*Full *Union	0, 0, 0, 0, 0, 0, 1	*0*	*1*	*0*

The full ANDing order weights were calculated by

ANDness(w)=1n−1∑r(n−r)wr
 MathType@MTEF@5@5@+=feaafiart1ev1aaatCvAUfKttLearuWrP9MDH5MBPbIqV92AaeXatLxBI9gBaebbnrfifHhDYfgasaacH8akY=wiFfYdH8Gipec8Eeeu0xXdbba9frFj0=OqFfea0dXdd9vqai=hGuQ8kuc9pgc9s8qqaq=dirpe0xb9q8qiLsFr0=vr0=vr0dc8meaabaqaciaacaGaaeqabaqabeGadaaakeaacqWGbbqqcqWGobGtcqWGebarcqWGUbGBcqWGLbqzcqWGZbWCcqWGZbWCcqGGOaakcqWG3bWDcqGGPaqkcqGH9aqpdaWcaaqaaiabigdaXaqaaiabd6gaUjabgkHiTiabigdaXaaadaaeqaqaaiabcIcaOiabd6gaUjabgkHiTiabdkhaYjabcMcaPiabdEha3naaBaaaleaacqWGYbGCaeqaaaqaaiabdkhaYbqab0GaeyyeIuoaaaa@49BA@

where *n *is the number of criteria in the MCE, *r *is the position of each criterion, and *w*_*r *_is the importance assigned to the particular criterion, *r *[30,39]. Table 2 lists the order weights that were assigned to the seven SES variables selected by the MHOs. For the full *intersection *of the order weights the first ranked SES criterion is multiplied by a value of 1 and the remaining SES criteria are multiplied by 0. Subsequent weights were gradually scaled across all SES criteria, with a weight of 0.142 assigned to each SES variable for a full trade-off of the order weights. Seven deprivation indices were constructed in total, three of which were synonymous with traditional OWA risk-adverse models, which were constructed from order weights from the full *intersection *to the trade-off model. The fourth index was a complete trade-off between the local and global weights. The remaining indices were synonymous with traditional OWA risk-seeking models, which were constructed using the weights between the trade-off and full *union *model.

The full *union *OWA model is the inverse of full *intersection *model and is calculated by

*ORness *= 1 - *ANDness*

The level of trade-off between the local and global weights is calculated from the same weights and variables used to construct the ANDness model by

TradeOff(w)=1−[n∑r(wr−1/n)2n−1]0.5
 MathType@MTEF@5@5@+=feaafiart1ev1aaatCvAUfKttLearuWrP9MDH5MBPbIqV92AaeXatLxBI9gBaebbnrfifHhDYfgasaacH8akY=wiFfYdH8Gipec8Eeeu0xXdbba9frFj0=OqFfea0dXdd9vqai=hGuQ8kuc9pgc9s8qqaq=dirpe0xb9q8qiLsFr0=vr0=vr0dc8meaabaqaciaacaGaaeqabaqabeGadaaakeaacqWGubavcqWGYbGCcqWGHbqycqWGKbazcqWGLbqzcqWGpbWtcqWGMbGzcqWGMbGzcqGGOaakcqWG3bWDcqGGPaqkcqGH9aqpcqaIXaqmcqGHsisldaWadaqaamaalaaabaGaemOBa42aaabeaeaacqGGOaakcqWG3bWDdaWgaaWcbaGaemOCaihabeaakiabgkHiTiabigdaXiabc+caViabd6gaUjabcMcaPmaaCaaaleqabaGaeGOmaidaaaqaaiabdkhaYbqab0GaeyyeIuoaaOqaaiabd6gaUjabgkHiTiabigdaXaaaaiaawUfacaGLDbaadaahaaWcbeqaaiabicdaWiabc6caUiabiwda1aaaaaa@5418@

The OWA scores assigned to the census units were reclassified into quintiles. The least deprived areas were assigned a value of 1 and the most deprived socio-economic areas assigned a value of 5.

To test the robustness of the OWA weights that were assigned to the original MHO survey responses we examined the prevalence scores were against the *Socio-economic Factor Index *(SEFI) deprivation index [18]. Briefly, the SEFI was constructed using a Factor Analysis on seven socio-economic variables taken from the Census and designed to measure area socio-economic inequality throughout urban and rural regions of Manitoba. The SEFI variables included an age dependency ratio of the population 65 and over, the proportion of single parent and female single parent households, female participation in the labour force, the area unemployment rate, and the proportion of residents with a minimum high school diploma. We made minor changes to the original SEFI index constructed by Frohlich and Mustard due to data limitations in the 2001 Census. The education component was measured using the percentages of people aged 15 years and over not the age specific rates of the original SEFI index. Due to data availability for unemployment data a factor analysis was calculated for only two age breakdowns (15–24, and 25 and over) rather than the original four age-group breakdowns.

## Results & discussion

**Figure 1:**
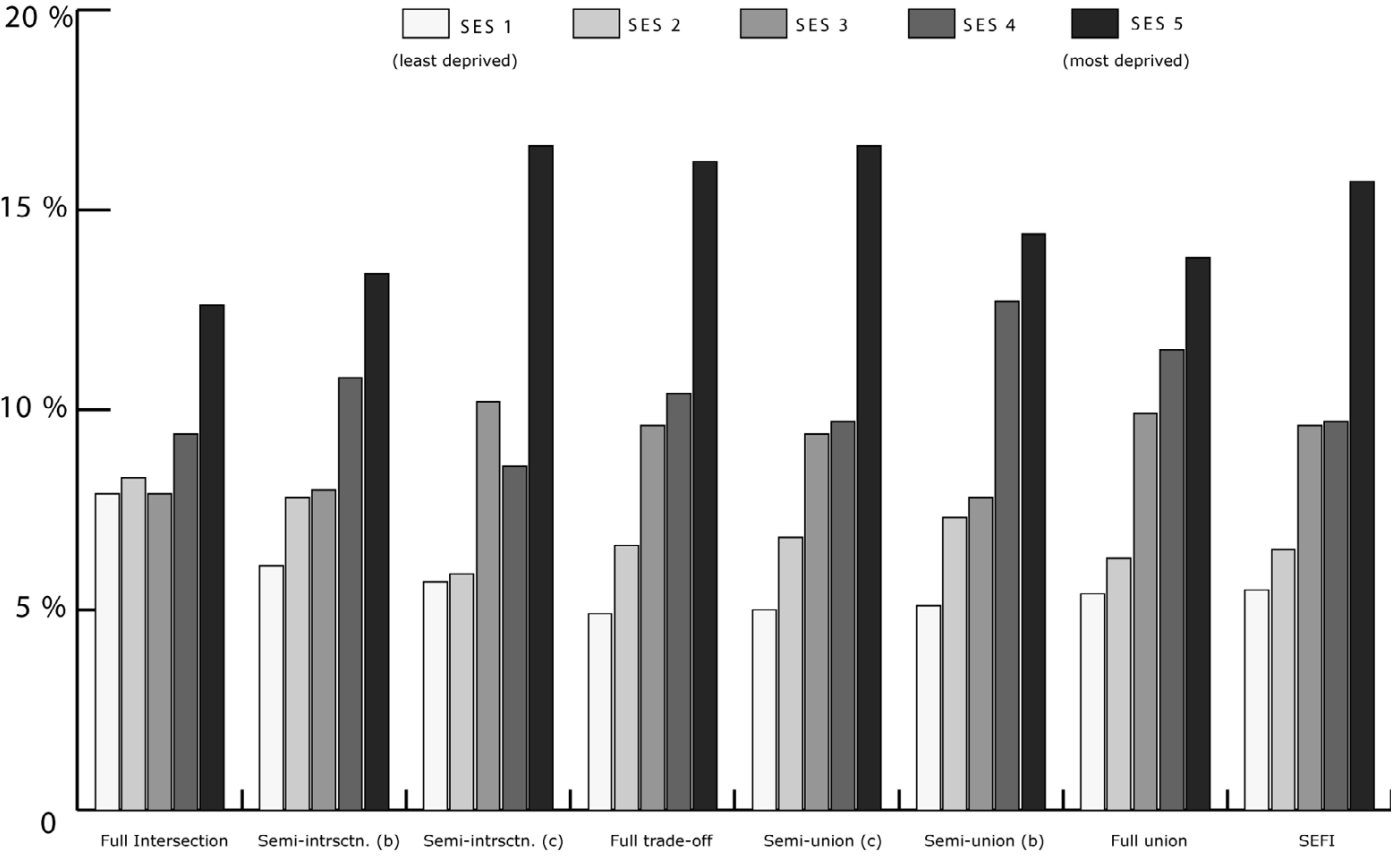
Prevalence scores of self rated health by neighbourhood SES. Quintile 1 in the Semi-Intersection (c); Trade-Off; Semi-Union (c), Semi-Union (b), and Full Union models have CV values between 16.6% and 33.3% which is considered marginal according to Statistics Canada data quality guidelines. Prevalence scores from the SEFI index were originally published in the following paper [54].

**Figure 2:**
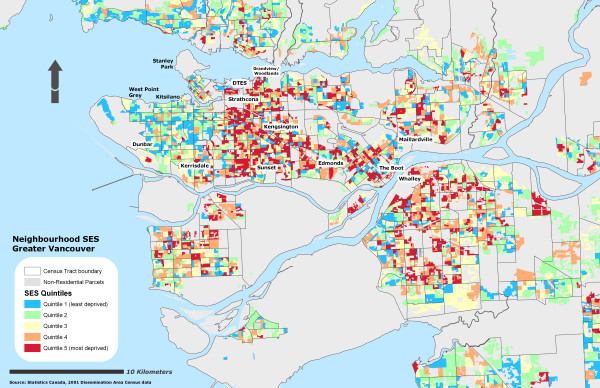
Vancouver CMA SES quintile rankings – OWA full intersection scenario.

**Figure 3:**
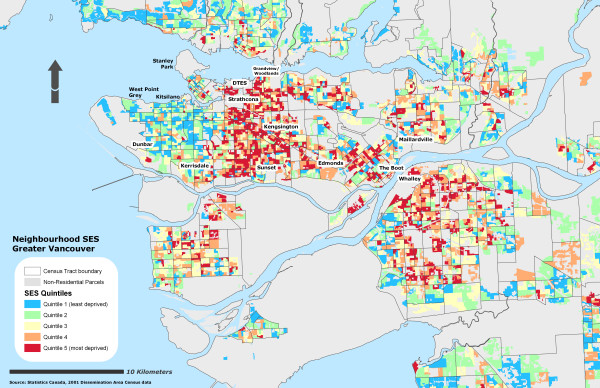
Vancouver CMA SES quintile rankings – OWA semi-intersection (b) scenario.

**Figure 4:**
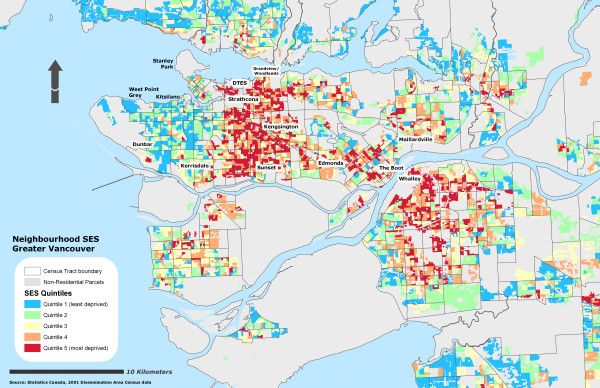
Vancouver CMA SES quintile rankings – OWA semi-intersection (c) scenario.

**Figure 5:**
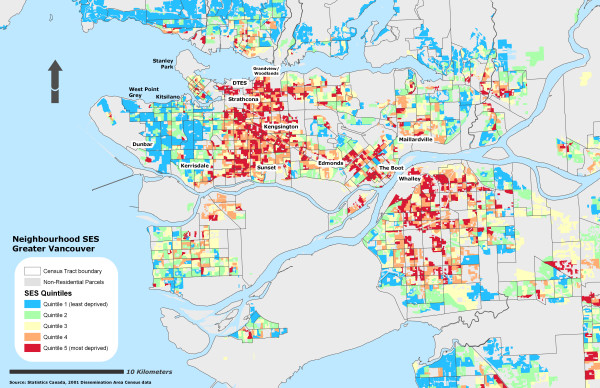
Vancouver CMA SES quintile rankings – OWA full trade-off scenario.

**Figure 6:**
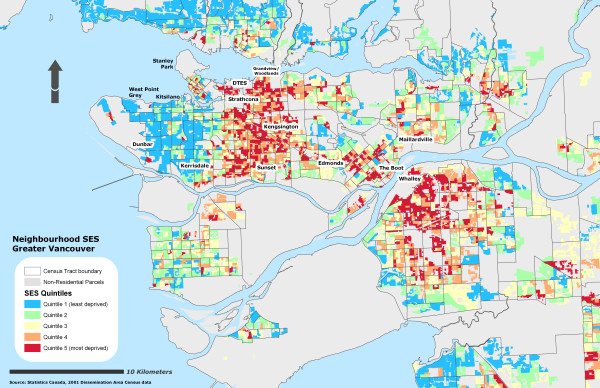
Vancouver CMA SES quintile rankings – OWA semi-union (c) scenario.

**Figure 7:**
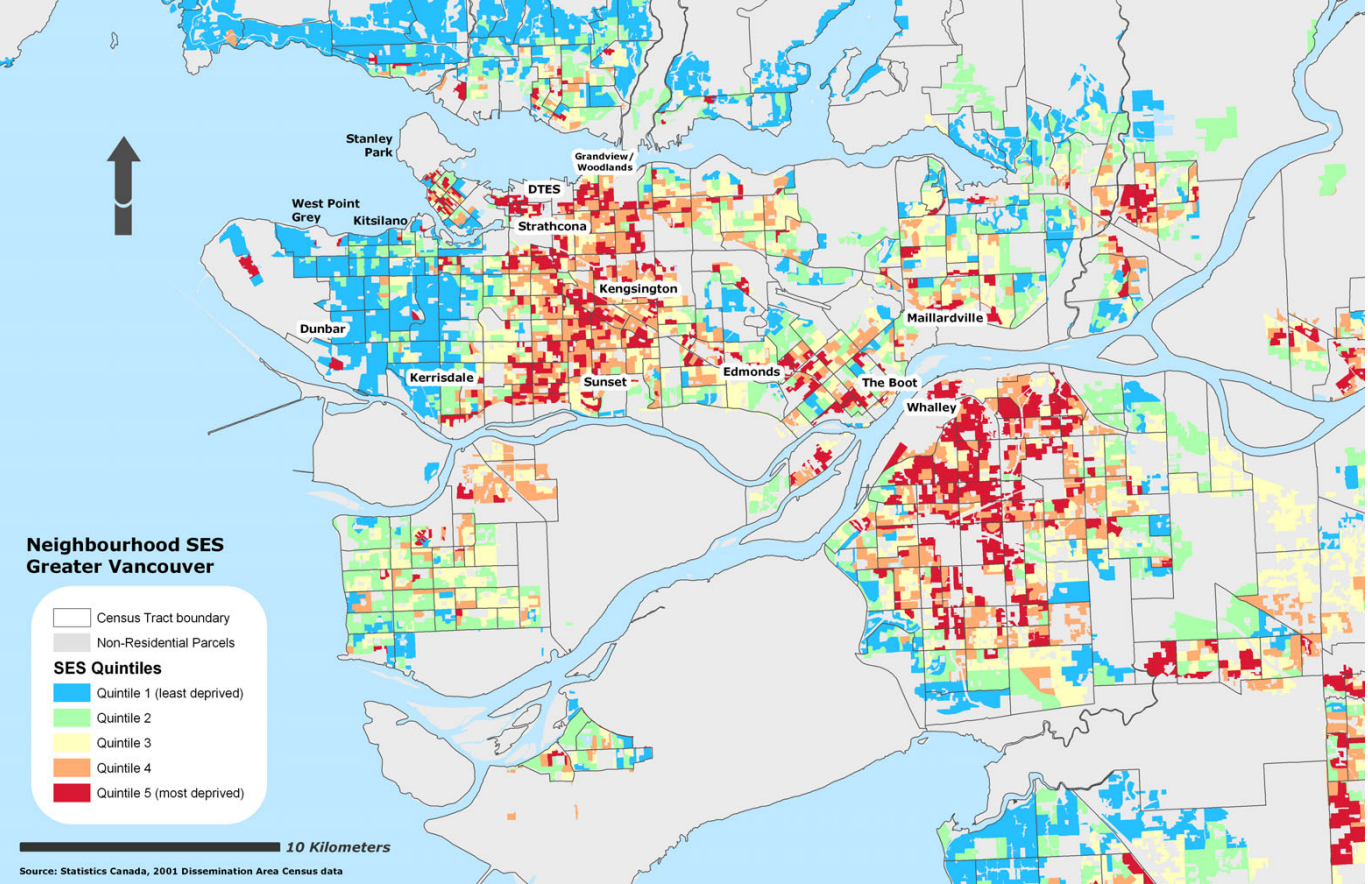
Vancouver CMA SES quintile rankings – OWA semi-union (b) scenario.

**Figure 8:**
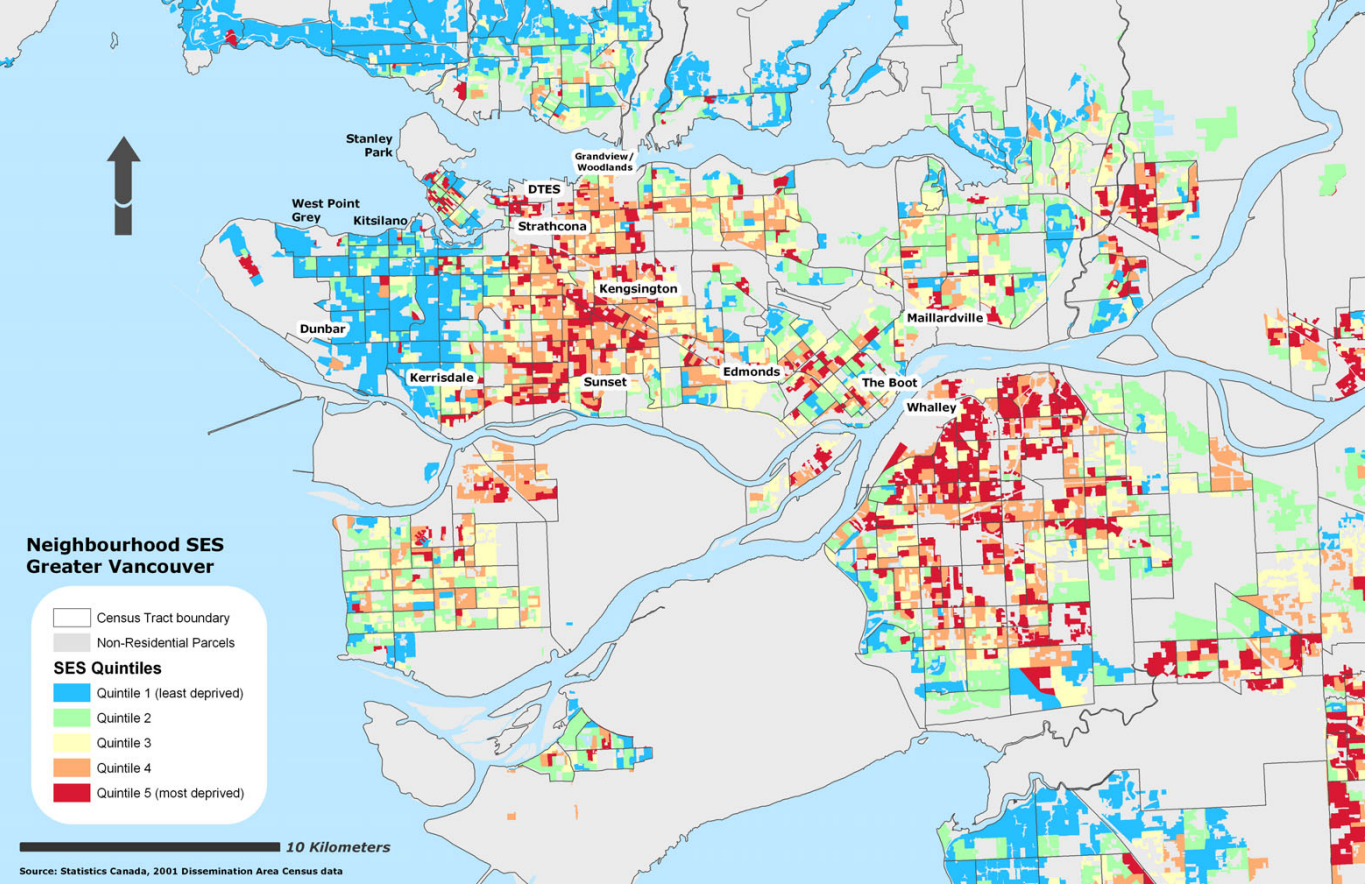
Vancouver CMA SES quintile rankings – OWA full union scenario.

**Figure 9:**
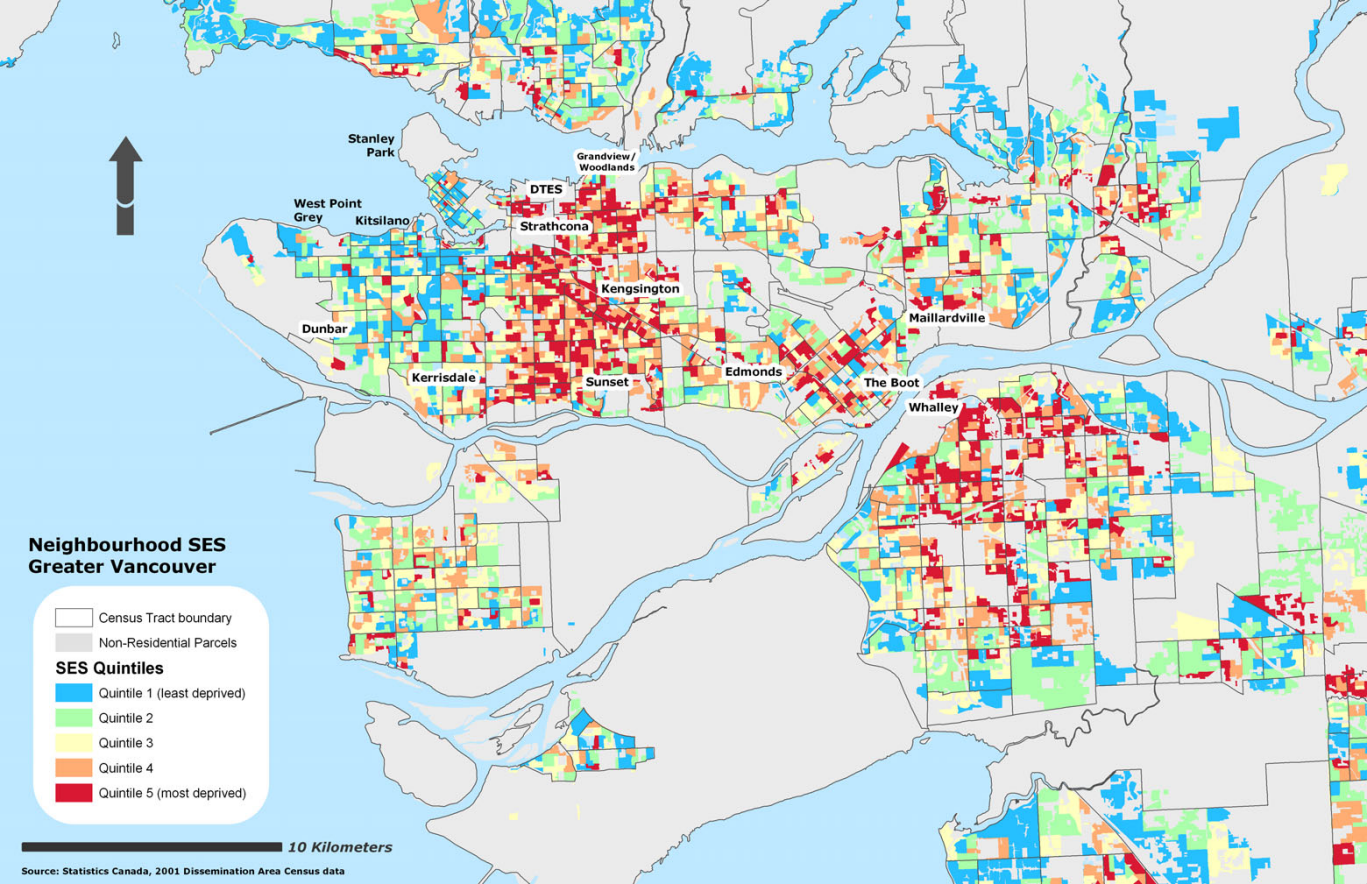
Vancouver CMA SES quintile rankings – SEFI deprivation index.

Due to the sensitivity of the health data, self-rated health records were aggregated into their corresponding Dissemination Area and used as a marker representing instances of reporting 'fair or poor health' self-rated health for the individual DA. To further protect individual confidentiality, the specific DA boundaries where the individuals reside are suppressed. 53% of the DAs had at least one resident who completed the survey (n = 3879) and an average of 2 residents per DA. Figure 1 illustrates the different OWA scenarios associated with the reporting 'fair or poor' self-rated health. All CV values for the prevalence scores at the DA level using the OWA indices were statistically significant (± 95% CI). All indices, on average, show a step-wise gradient across neighbourhood SES and self-rated health. Only two of the risk-adverse OWA *intersection *weighted indices contained discrepancies between SES quintile ranking and health rating, but the overall separation between the least and most deprived SES quintile and self-rated health scores remained. Maps of the Vancouver CMA for each OWA scenario are provided in figures 2, 3, 4, 5, 6, 7, 8, 9.

It is important to recognize that both the full *intersection *and *union *OWA models were constructed using a single itinerant SES variable rather than the amalgamation of the variables selected by the MHOs. The full *intersection *scenario allotted the most control over the global weights and constructed from the SES variable with the lowest value. The full *union *model is still assessed using the order weights although in this scenario the model maximizes the global weights using the largest SES variable. Table 5 lists the influence of the order weights on the SES variables within each scenario.

**Table 5:** The percentage of representation of individual SES indicators for each OWA weighting scenario.

	----- Greater Decision Uncertainty -----				----- Lower Decision Uncertainty -----		
			
**SES Variable (original MHO rank)**	*Full *Intersection*	*AND *(b)	*AND *(c)	Trade-off	*OR *(c)	*OR *(b)	*Full *Union
Average Income (6^th^)	--	--	99%	100%	99%	99%	72%
Home Ownership (5^th^)	7%	62%	99%	100%	99%	58%	6%
Lone Parent Families (4^th^)	11%	81%	91%	100%	91%	32%	--
Without High School Education (1^st^)	10%	75%	99%	100%	99%	49%	--
With University Degree (3^rd^)	--	2%	99%	100%	99%	99%	23%
Unemployment Rate (2^nd^)	1%	70%	99%	100%	99%	59%	--
Employment Ratio (7^th^)	51%	88%	88%	100%	88%	3%	--

*column sums to less than 100% do to normalization of SES variables

A step-wise gradient in self-rated health is revealed within the full *intersection *model, although the gap between the least and most deprived SES quintiles is the narrowest of all seven OWA indices at 4.7%. Closer examination of the full *intersection *modeling scenario revealed that nearly 60% of the SES quintiles were represented by the employment ratio in the Vancouver CMA. Several implications can be drawn from this finding. Unemployment trends, on average, have been declining in the CMA over the course of the past two decades so it is not so surprising to find that its significance is somewhat waning. Underemployment rather than employment ratios may provide a more significant relationship to self-rated health. Interestingly, this order weight is synonymous with the rank position of employment ratios in the global weights assigned by the MHOs. To that effect, the order weights were reflective of the significance placed on employment ratios by the MHOs towards characterizing relative health outcomes.

When the order weights were maximized in the full *union *scenario average income was the single most representative SES variable, representing nearly 75% of the DAs against the self-rated health data. In contrast with employment ratios, incidence of low and below average income rates are heavily concentrated within the CMA, especially in neighbourhoods surrounding the Downtown Eastside, Edmonds and south along the waterfront in New Westminster, and encompassing Whalley in North Surrey. Interestingly, only the employment ratio was ranked lower than average income by the MHOs. With the addition of the local weights onto average income, prevalence rates associated with the *least *deprived quintiles fell from 7.9% to 5.4%.

Within the *most *deprived SES quintiles both the full *union *and *intersection *models produced similar scenarios, but with prevalence rates slightly higher using the full *union *model at 13.8%. Overall, both of the extremity weighting scenarios revealed a social gradient in self-rated health in the Vancouver CMA. However, when greater decision uncertainty is placed on the MHO variable rankings the prevalence scores between self-rated health and neighbourhood SES are reduced. Although the full *intersection *model does not furnish as robust step-wise gradient in overall self-rated health as its antipode, it does provide a spatial filter for evaluating variable nuance of the MHO rankings.

SES variables were included into the risk-adverse and risk-seeking OWA models until a full trade-off of the local and global weights was obtained. Until a combination of all seven SES variables were introduced into the risk-adverse OWA models, however, area SES was primarily analyzed by applying lower weighting schemas to variables previously ranked amongst the *most *significant by the MHOs. In the full and semi-*intersection *model (b) unemployment and average income reaffirm their positive and negative extremes in addition to under weighting the original significance assigned to secondary and higher education. In the full trade-off OWA index, however, the order weights exert the least amount of control over the original MHO variable rankings. In this case, the results indicate that the steepest of all seven step-wise gradients between neighbourhood SES and self-rated health are found when the original responses and frequency weights assigned by British Columbia's MHOs are interpreted with minimum decision uncertainty and control. The method illustrated here is a means of assigning uncertainty to expert group weights in the event that validation and objective assessments of their selection is necessary.

On average, both the SEFI and OWA indices produced nearly identical SES classifications throughout greater Vancouver. Only the OWA indices that utilized all seven of the indicator variables produced wider social gradients in self-rated health outcomes. The step-wise gradient revealed when using the SEFI index ranged from 5.2% in the least deprived quintile to 15.7% in the most deprived quintile, which was slightly narrower than the 4.9% and 16.2% range from the MHO weighted indicators. As expected, Spearman rank correlation coefficient between the SEFI and seven OWA indices revealed that the indices were more similar than dissimilar. Both utilize indicators for lone parent families, area unemployment rates, and secondary educational attainment. The correlation coefficient was the lowest between the OWA indices nearer the full *intersection *and *union *(0.469 p < 0.01, 0.544 p < 0.01) and the strongest when the OWA index was constructed from all seven variables (0.772 p < 0.01). This drop in correlation likely stems from the high proportion of single variable representation as the OWA weights near the full Boolean *intersection *and *union*.

Some attenuation between SES quintile rankings and prevalence of reporting 'fair or poor' self-rated health were observed between the original MHO weighted index and the OWA constructed indices. The original web-survey generated prevalence scores ranged from 4.0% in the least deprived quintile to 17.3% in the most deprived quintile, with rates rising step-wise across SES quintiles 2 – 4 from 8 – 9.8% at the DA spatial extent. The differences between the original and augmented MHO weights were small, with the gradient between least and most deprived quintiles attenuating by 1.0% in the least deprived quintiles and 0.6% in the most deprived SES quintiles. The variation in prevalence scores likely reflects smoothing of the indicator variables when multiplied by the constant averaging weight of 0.142. The similarity between the original MHO index and the OWA and SEFI weighted indices points to the well-known observation that different variable weights yield different results. As the OWA method was originally designed to validate the MHO response scores these results also suggest that the MHOs are well-versed in the conditions that characterize health inequalities within British Columbia.

Although our analysis was not age-adjusted by individual SES variables contextual effects of community socio-economic characteristics are well-known indicators of population health independent of individual SES [52,53]. Moreover, scenario modeling of survey response scores should not obscure the fact that socio-economic status remains positively related to health status throughout the Vancouver CMA regardless of deprivation index. The relationship between neighborhood SES and prevalence of reporting 'fair or poor' self-rated health is equally pronounced when assessed by provincial MHOs as when evaluated using variations of Principal Component Analysis, which suggests that MHOs can play a valuable role in quantitative evaluations of population health. Variations between both the SEFI and OWA indices suggests that the dissimilar variables used by both indices are equally important indicators of the conditions that tend to increase social gradient in health, but that developing local socio-economic deprivation indicators may be a more appropriate strategy.

The location of individual self-rated health responses are concealed to protect anonymity, but given the prevalence scores between self-rated health and the SES variables selected by the MHOs it would not be surprising to find greater morbidity and mortality levels in these areas. Evaluating socioeconomic inequalities using OWA offers a flexible means of assessing systematic differences in neighbourhood SES as viewed by provincial MHOs and provides a mechanism for validating expert opinion using of local and globally defined weighting scenarios.

## Conclusion

The approach presented in this research is methodologically and conceptually distinct from traditional deprivation index construction. Unlike previous survey-based deprivation indices, the OWA index weights are simultaneously influenced by the experts and the data. This is a new method that may diminish the historic tension of incorporating highly subjective user assigned weights into spatial analysis. The local weights also provide leverage for controlling the level of uncertainty in the interpretation and weighting of the SES variables selected by the MHOs. Future research will focus on the use of OWA as a new vantage point for generalizing how variable weights influence one another (i.e. effect of education on income). This may offer a more robust understanding of neighbourhood variations in SES.

## Competing interests

The author(s) declare that they have no competing interests.

## Authors' contributions

NB drafted the manuscript, performed the statistical analysis, and constructed the maps and tables. NS and MH conceived early segments of the study and helped draft the original manuscript. All authors read and approved the final manuscript.

## Disclaimer

This analysis was based on the Statistics Canada master file CCHS (Cycle 2.1) which contains anonymized data collected in 2003. All computations were prepared by the authors and conducted at the British Columbia Interuniversity Research Data Centre, University of British Columbia. The responsibility for the use and interpretation of these data is solely that of the authors. The opinions expressed in this paper are those of the authors and do not represent the views of Statistics Canada.
